# The Prevalence of Metabolic Disease Multimorbidity and Its Associations With Spending and Health Outcomes in Middle-Aged and Elderly Chinese Adults

**DOI:** 10.3389/fpubh.2021.658706

**Published:** 2021-05-03

**Authors:** Yang Zhao, Puhong Zhang, John Tayu Lee, Brian Oldenburg, Alexander van Heusden, Tilahun Nigatu Haregu, Haipeng Wang

**Affiliations:** ^1^Stroke and Women and Children Health Program, The George Institute for Global Health at Peking University Health Science Center, Beijing, China; ^2^Non-communicable Disease Unit, The Nossal Institute for Global Health, The University of Melbourne, Melbourne, VIC, Australia; ^3^WHO Collaborating Centre on Implementation Research for Prevention and Control of Non-communicable Diseases, The University of Melbourne, Melbourne, VIC, Australia; ^4^Faculty of Medicine, University of New South Wales, Sydney, NSW, Australia; ^5^Centre for Health Management and Policy Research, School of Public Health, Cheeloo College of Medicine, Shandong University, Jinan, China; ^6^NHC Key Laboratory of Health Economics and Policy Research (Shandong University), Jinan, China

**Keywords:** metabolic disease, multimorbidity, healthcare spending, health outcome, Chinese adults

## Abstract

**Objective:** Metabolic diseases have been a clinical challenge worldwide and a major public health issue. Very few studies from China investigated the impact of metabolic multimorbidity on healthcare and health outcomes at the national level. This study aims to examine the association of metabolic multimorbidity with health service utilization, spending, functional and mental health.

**Materials and Methods:** This is a nationally representative cross-sectional study, utilizing the data from the China Health and Retirement Longitudinal Study in 2015, including 11,377 participants aged 45 years and older. Multivariable regression models were used to assess the association of metabolic multimorbidity with healthcare, out-of-pocket expenditure (OOPE), the activities of daily living (ADL) limitation, the instrumental activities of daily living (IADL) limitation, and depression.

**Results:** Overall, 30.50% of total participants had metabolic multimorbidity in 2015 in China. Compared with single disease, metabolic multimorbidity were associated with the number of outpatient visits [incident rate ratio (IRR) = 1.30, 95% CI = 1.05, 1.62] and days of inpatient care (IRR = 1.52, 95% CI = 1.28, 1.81). Metabolic multimorbidity was positively associated with the OOPE on outpatient care (coefficient = 82.99, 95% CI = 17.70, 148.27) and physical functional difficulties, including ADL limitation (odds ratio = 1.36, 95% CI = 1.18, 1.57).

**Conclusions:** Metabolic multimorbidity is associated with higher levels of health-care service use, greater expenditure for outpatient care, and more difficulties in ADL among Chinese adults. China's health-care systems need to shift from single-disease models to new financing and service delivery models to effectively manage metabolic multimorbidity.

## Introduction

Metabolic diseases have been a major public health issue and a clinical challenge worldwide, which is linked with the increased risk of cardiovascular diseases (1, 2) and all-cause mortality (3, 4). The prevalence of metabolic multimorbidity (defined as presence of two or more chronic conditions including hypertension, dyslipidaemia, diabetes, hyperuricemia and central obesity) is increasing rapidly. Recently, the Emerging Risk Factor Collaboration of 91 cohort studies showed that a particular form of cardiometabolic multimorbidity, was associated with a risk of death substantially greater than that for each of these diseases on their own. For example, at age 60 years, people with one cardiometabolic disease had a life expectancy 6–10 years shorter than those with no such disease, whereas people with cardiometabolic multimorbidity had a life expectancy shorter by up to 15 years (5). Over the past decade, a rapid increase was found in the number of individuals suffering from metabolic syndrome multimorbidity in China and low-and middle income countries (LMICs) (6–8).

Multimorbidity is associated with higher healthcare utilization, worse health status and depression in European countries, challenging the single-disease framework by which most of healthcare is configured (9). While there have been many studies conducted in high-income countries (HICs) on the impacts of chronic disease multimorbidity (10–12), this topic is still an emerging area of research inquiry in LMICs. Currently, only a couple of small studies in certain parts of China have examined this issue (13, 14), such as a study focused on Guangdong which looked at the health service utilization arising from multimorbidity of 162,464 subjects (15). Emerging evidence exists about the impact of single chronic condition alone. However, evidence is sparse about the economic and financial impact among people who have two or more metabolic conditions concurrently. No current study from China has estimated the impact of metabolic multimorbidity on functional limitation and mental health at the national level (16, 17). This study aims to systemically examine the association of metabolic multimorbidity with healthcare utilization, out-of-pocket expenditure (OOPE), functional health and depression, using nationally representative population-based data.

## Materials and Methods

### Designated Population and Sample

This is a nationally representative cross-sectional study, using the newest round of data from the China Health and Retirement Longitudinal Study (CHARLS) conducted in 2015. CHARLS is a biennial survey conducted by the National School of Development at Peking University, which aimed to be representative of Chinese residents aged 45 years and older. The data was collected in a survey in which four-stage, stratified, cluster sampling was used to select eligible individuals (18). Brie?y, 150 counties were selected, proportional to population size. Then three villages/communities were selected from each county as primary sampling units (PSUs). In each of the 450 PSUs, 80 households were randomly selected. In each household, persons aged 45 years and over, as well as their spouses, were interviewed using structured questionnaires. The main questionnaire includes information on basic demographics, health status and functioning, healthcare and insurance, work, retirement and pensions, income and consumption, household assets, and several biomarkers. Written informed consent was obtained from all participants. CHARLS received ethics approval from the Peking University Biomedical Ethics Review Committee (Ref. no. IRB00001052-11015) in 2011 (18).

The total sample size of the CHARLS baseline survey was 17,708 individual respondents. Ongoing follow-up surveys were conducted once every 2 years. For this study, we identified 13,420 respondents with blood test and biomarker information. After removing respondents aged below 45 years and those individuals with missing values of dependent or independent variables, our final sample consisted of 11,377 respondents accounting for 84.8% of those without loss-to-follow-up.

### Definition of the Metabolic Diseases

In this study, we counted the number of chronic diseases for each participant, identifying those with multimorbidity (19, 20). Hypertension was defined as systolic blood pressure ≥140 mmHg and/or diastolic blood pressure ≥90 mmHg, and/or being on antihypertensive medication for raised blood pressure (21). Diabetes was defined by (1) a fasting plasma glucose level of ≥126 mg/dL (7.0 mmol/L); and/or (2) HbA1c concentration of ≥6.5%; and/or (3) being insulin treatment and/or taking medication for raised blood sugar (22). Dyslipidaemia was defined by (1) total cholesterol (TC) ≥ 240 mg/dL (6.22 mmol/L); and/or (2) low-density lipoprotein cholesterol (LDL-C) ≥ 160 mg/dL (4.14 mmol/L); and/or (3) high-density lipoprotein cholesterol (HDL-C) <40 mg/dL (1.04 mmol/L); and/or (4) triglyceride (TG) ≥ 200 mg/dL (2.26 mmol/L); and/or (5) taking anti-dyslipidaemia medication (23). Hyperuricemia was defined as a blood uric acid concentration > 7.0 mg/dL for men and 6.0 mg/dL for women (24). Central obesity was defined as a waist circumference > 90 cm for men and 85 cm for women in participants with Body mass index (BMI) ≥ 30 kg/m^2^ (25).

### Outcome Variables

Respondents were asked about their utilization of healthcare services, including the frequency of outpatient visits and days of inpatient care: “How many times did you visit a general hospital, specialized hospital, clinic or other medical facilities for outpatient care in the past month? and How many days did you stay in hospital over the past year?” CHARLS also collected the information on how much respondents paid in total and how much out-of-pocket (deducting the reimbursed expenses) for their outpatient visits during the last month and inpatient care during the last year.

Functional health was assessed by activities of daily living (ADL) limitation and instrument activities of daily living (IADL) limitation (26). The ADL includes six activities: bathing, dressing, feeding oneself, using the toilet, getting in or out of bed, and controlling urination and defecation. Answers were categorized as: “can do it by myself,” “have some difficulties,” “need help,” and “cannot do it.” The IADL refers to difficulty in doing household chores, cooking, shopping, making telephone calls, taking medications, and managing finances. Binary variables of ADL/IADL were constructed, and ADL/IADL disability was defined as having difficulty in one or more ADL/IADL items. This binary coding of ADL/IADL variables was also used as dependent variables in the multivariate regression analysis.

In terms of mental health, depression was assessed by the 10-item Center for Epidemiologic Studies Depression Scale (CES-D 10) (27), which has been identified as a valid, reliable, and useful mental health assessment tool for those aged 60 and above in China (28). The answers of the CES-D 10 include 4 options: (1) rarely, (2) some days (1–2 days per week), (3) occasionally (3–4 days per week), (4) most of the time (5–7 days per week). The participants' answers were recorded as 0 (rarely) to 3 (most of the time) for the negative questions in this study. For two positive questions, the items were reversed as 3 (rarely) to 0 (most of the time). The total scores of the CESD-10 range from 0 to 30. In this study, a binary variable of mental health was also constructed by defining an individual whose CESD-10 score was < 10 as having depression symptoms.

### Statistical Analysis

The negative binomial regression models were applied to investigate the association of multimorbidity with the frequency of outpatient visits and inpatient care. Covariates included age, gender, marital status (married and partnered, unmarried and others), education (illiterate, primary school and below, secondary school, college and above), residence place (rural, urban), geographical region (east, central and west), economic status quartiles (yearly per capita household consumption expenditure), and health insurance status (yes, no). Linear regression models were used to examine the relationships between multimorbidity and outpatient and inpatient care OOPE. Multivariable logistic regression models were used to estimate the association of multimorbidity with functional limitation and depression. We also performed sensitivity analyses for the impacts of multimorbidity on health service by using Poisson regression models and for the impacts on OOPE by using generalized linear models with a logarithm transfer.

For the negative binomial regression analysis, the incident rate ratio (IRR) were reported with results of 95% confidence intervals (CI) included in the Appendix. For linear regression models, we reported the coefficient (β) and 95% CI. For the logistic regression analysis, the adjusted odds ratio (AOR) and 95% CI were reported. Descriptive analysis of prevalence of multimorbidity and regression analysis were weighted to account for the multi-stage PPS design of CHARLS. All statistical analyses were conducted using STATA 15.0. *P* < 0.05 were considered as statistically significant.

## Results

Our analysis included data from 11,377 participants. The mean age of respondents was 60.29 years in 2015. Among the participants, 52.62% were women, 43.10% of the participants were illiterate, 63.18% were residing in rural areas and 82.23% were enrolled in social health insurance schemes. The prevalence of metabolic multimorbidity was 32.47% and increased with age, ranging from 24.61% in those aged 45–54 years, and 41.62% for those aged ≥ 75 years. People living in urban areas and those covered by social health insurance were suffering from a higher percentage of multimorbidity in China, compared with rural residents and individuals without health insurance (Table 1).

**Table 1:** Characteristics of participants, the prevalence of single condition, and metabolic multimorbidity.

**Variables**	***N*** **(Percentage)**^**a**^		**Prevalence of single disease**^**b**^ **(95% CI)**			**Prevalence of multimorbidity (95% CI)**		
All	11,377	100.00	32.97	31.63	34.33	32.47	30.9	33.98
**Age, years**								
45–55	3,633	31.93	30.90	28.32	33.60	24.61	21.84	27.60
55–65	4,084	35.90	32.81	30.77	34.91	34.47	32.01	37.01
65–75	2,722	23.93	34.93	32.39	37.54	37.88	35.39	40.43
≥75	938	8.24	36.81	32.70	41.12	41.62	36.86	46.55
**Gender**								
Male	5,390	47.38	34.04	32.04	36.11	33.99	31.75	36.31
Female	5,987	52.62	31.94	30.19	33.75	31.01	29.09	33.00
**Marital status**								
Married and partnered	9,918	87.18	32.72	31.26	34.22	32.14	30.49	33.82
Unmarried and others	1,459	12.82	34.66	31.73	37.70	34.75	31.87	37.75
**Education level**								
Illiterate	4,903	43.10	33.32	31.72	34.95	32.01	30.12	33.97
Primary school	3,110	27.34	31.62	29.02	34.34	32.48	29.60	35.49
Secondary school	2,256	19.83	34.26	30.90	37.79	30.99	28.32	33.78
College and above	1,108	9.74	32.68	28.45	37.21	36.11	30.25	42.41
**Residence place**								
Urban	4,189	36.82	31.41	29.00	33.92	38.08	35.33	40.92
Rural	7,188	63.18	34.42	33.22	35.64	27.24	26.12	28.38
**Region**								
East	4,268	37.51	33.32	30.72	36.03	34.92	31.92	38.04
Central	4,370	38.41	33.25	31.54	35.01	32.01	30.30	33.76
West	2,739	24.07	31.83	29.87	33.86	28.63	26.70	30.64
**PCE, RMB**								
<5,000	4,554	40.03	32.44	30.50	34.44	30.48	28.12	32.94
≥5,000	3,462	30.43	33.37	30.96	35.86	34.93	32.43	37.52
Missing	3,361	29.54	33.18	30.60	35.86	32.24	29.43	35.19
**Social health insurance**								
No	2,022	17.77	32.82	29.24	36.60	26.90	23.72	30.33
Yes	9,355	82.23	33.00	31.60	34.43	33.82	32.18	35.50

a *N and percentages were based on study samples (unweighted)*;

b *Weighted prevalence of single disease and metabolic multimorbidity*.

Table 2 showed the prevalence of main metabolic diseases and multimorbidity across gender and age group. Among the middle-aged and older population, more than a third of Chinese adults suffered from hypertension and hyperlipidaemia in 2015. The prevalence of diabetes, hyperuricemia and concentric obesity was 19.75, 13.70, and 4.92%, respectively. The prevalence of hypertension and metabolic multimorbidity increased with age among both males and females, but concentric obesity decreased with age in female populations. For diabetes and hyperuricemia, the senior older group (aged ≥ 75 years) suffered from the highest prevalence.

**Table 2:** The proportion of metabolic diseases and multimorbidity among Chinese adults by gender and age group.

**Metabolic disease**	**Total**		**Male**				**Female**			
	***N***	**%**	**45–55**	**55–65**	**65–75**	**≥75**	**45–55**	**55–65**	**65–75**	**≥75**
**Single disorder**										
Hypertension	4,269	36.90	26.10	40.25	48.18	49.41	21.50	35.16	49.24	63.73
Hyperlipidaemia	4,001	36.45	37.51	41.05	36.04	28.21	30.18	38.20	41.16	31.00
Diabetes	2,153	19.75	14.13	22.88	19.75	27.15	11.21	22.61	25.97	29.50
Hyperuricemia	1,299	13.70	18.14	15.37	14.50	20.78	10.15	8.87	12.75	19.49
Concentric obesity	588	4.92	4.56	3.36	2.64	2.84	7.63	6.41	4.89	2.82
**Multimorbidity cluster**									
Overall multimorbidity	3,470	32.47	28.38	36.77	35.92	38.52	21.32	32.31	39.97	44.89
Hypertension multimorbidity	2,600	22.95	17.09	25.34	29.37	25.03	11.61	23.49	32.84	37.75
Hyperlipidaemia multimorbidity	2,627	24.56	21.69	29.79	26.81	22.57	17.08	25.03	29.79	27.26
Diabetes multimorbidity	1,676	15.85	11.99	18.79	15.21	22.16	7.87	17.73	21.10	26.89
Hyperuricemia multimorbidity	1,037	10.99	13.05	12.11	11.68	18.63	8.12	7.55	11.29	15.38
Concentric obesity multimorbidity	519	4.40	4.21	3.01	2.64	2.84	6.38	5.62	4.58	2.82

*Values are unweighted counts and weighted percentages unless otherwise indicated. Overall multimorbidity refers to any two or more metabolic conditions included in this study. Hypertension multimorbidity refers to hypertension with any one or more other metabolic conditions; Hyperlipidaemia multimorbidity refers to hyperlipidaemia with any one or more other metabolic conditions; Diabetes multimorbidity refers to diabetes with any one or more other metabolic conditions; Hyperuricemia multimorbidity refers to hyperuricemia with any one or more other metabolic conditions; Concentric obesity multimorbidity refers to concentric obesity with any one or more other metabolic conditions*.

Table 3 indicated that multimorbidity was positively associated with health service use. Compared with people with single metabolic disease, patients with multimorbidity were likely to report more frequent outpatient visits (IRR = 1.30) and days of inpatient care (IRR = 1.52). The days of inpatient care increased substantially with age. Female patients used both outpatient and inpatient healthcare service more frequently than male patients. Individuals with a higher economic level and those patients in economically underdeveloped regions had more days of hospitalization compared with their counterparts.

**Table 3:** The association of metabolic multimorbidity with the frequency of healthcare utilization.

**Variable (reference)**	**Number of outpatient visits**		**Days of inpatient care**		**OOPE for outpatient care**		**OOPE for inpatient care**	
	**IRR**	***P*-value**	**IRR**	***P*-value**	**β**	***P*-value**	**β**	***P*-value**
Multimorbidity (single disorder)	1.30	0.017	1.52	<0.001	82.99	0.013	108.43	0.707
**Age (45–59 years)**								
55–65	0.97	0.818	1.55	<0.001	−15.82	0.682	556.90	0.069
65–75	1.09	0.484	1.99	<0.001	3.11	0.939	1,069.96	0.050
≥75	1.11	0.609	2.58	<0.001	−95.19	0.102	709.60	0.024
Gender (male)	1.48	<0.001	1.22	0.031	47.19	0.039	498.08	0.161
Marital status (married)	0.98	0.845	0.95	0.624	60.30	0.459	−631.38	0.006
**Education level (Illiterate)**								
Primary school	0.98	0.876	1.12	0.266	36.39	0.263	663.58	0.149
Secondary school	0.92	0.546	1.17	0.243	12.45	0.719	211.25	0.456
College and above	1.01	0.963	0.93	0.657	−21.02	0.529	224.85	0.746
Residence place (urban)	1.21	0.107	1.07	0.460	98.08	0.007	−238.30	0.313
**Region (east)**								
Central	1.06	0.608	1.43	0.004	−5.20	0.884	−321.03	0.383
West	1.22	0.146	1.81	<0.001	−34.89	0.421	−303.85	0.319
**PCE (<5000 RMB)**								
≥5000 RMB	1.10	0.401	1.52	<0.001	128.44	<0.001	1,218.25	0.001
Missing	1.10	0.448	1.34	0.003	84.70	0.020	166.34	0.348
Social health insurance (none)	1.13	0.449	1.15	0.268	10.89	0.762	526.56	0.039

*Negative binomial regression models were used and adjusted for all socio-demographic covariates. IRR, incident rate ratio; CI, confidence interval; PCE, Per capita household annual consumption expenditure*.

**Figure 1:**
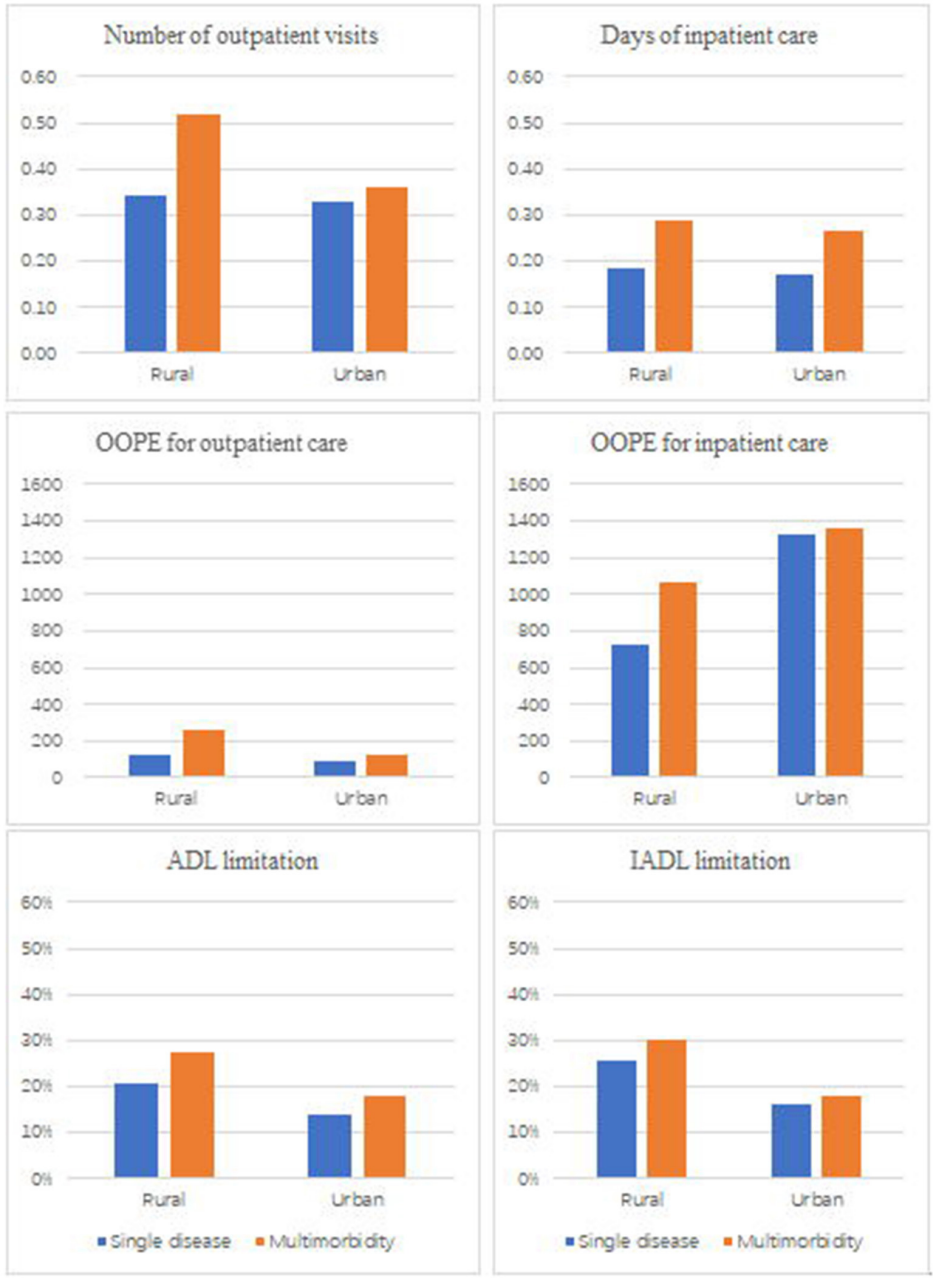
Frequency of healthcare, health expenditure, and functional limitation by the number of disease and residence place.

Table 3 also showed that the prevalence of metabolic multimorbidity had a positive relationship with healthcare expenditure. The out-of-pocket spending on outpatient care was higher for patients with multimorbidity than those with a single disorder (β = 82.99). OOPE on outpatient care was significantly higher among female patients and those living in rural areas than spending among the male and urban citizens (Figure 1). The affluent population was likely to spend more out-of-pocket money on outpatient care (β = 128.44) and hospitalization care (β = 1,218.2) than those in a lower economic level. There was no statistically significant association between multimorbidity and OOPE for inpatient care. People enrolled in health insurance were likely to spend more on hospitalization care (β = 526.56) than those individuals without health insurance.

Table 4 showed that multimorbidity was associated with a higher likelihood of having functional difficulties. Compared with people with single metabolic disease, patients with multimorbidity were more likely to have an ADL limitation (AOR = 1.36). Older patients, females, those people with lower education status, and living in rural areas and undeveloped regions were reported higher levels of ADL limitation, IADL limitation and depression than their counterparts. No statistically significant association was found between multimorbidity and depression.

**Table 4:** The association of metabolic multimorbidity with functional and mental health.

**Variable (reference)**	**ADL limitation**		**IADL limitation**		**Depression**	
	**AOR**	***P*-value**	**AOR**	***P*-value**	**AOR**	***P*-value**
Multimorbidity (single disorder)	1.36	<0.001	1.16	0.051	0.99	0.871
**Age (45–59 years)**						
55–65	1.47	0.023	1.61	0.003	1.59	<0.001
65–75	2.15	<0.001	2.50	<0.001	1.87	<0.001
≥75	2.94	<0.001	4.80	<0.001	0.99	0.940
Gender (male)	1.45	<0.001	1.90	<0.001	2.03	<0.001
Marital status (married)	1.11	0.392	0.93	0.508	1.31	0.006
**Education level (Illiterate)**						
Primary school	0.72	0.008	0.49	<0.001	0.81	0.033
Secondary school	0.62	<0.001	0.48	<0.001	0.87	0.294
College and above	0.37	<0.001	0.21	<0.001	0.43	<0.001
Residence place (urban)	1.37	0.011	1.46	0.001	1.78	<0.001
**Region (east)**						
Central	1.81	<0.001	1.73	<0.001	1.49	<0.001
West	1.46	0.006	1.70	<0.001	1.86	<0.001
PCE (<5000 RMB)						
≥5000 RMB	1.03	0.800	0.90	0.314	0.92	0.369
Missing	1.23	0.043	1.18	0.076	0.88	0.182
Social health insurance (none)	1.21	0.103	1.16	0.145	2.47	<0.001

*PCE, Per capita household annual consumption expenditure; AOR, Adjusted odds ratio; CI, confidence interval*.

In terms of sensitivity analyses, we found similar associations between metabolic multimorbidity and frequency of outpatient and inpatient care as well as OOPE (Supplementary Tables 1, 2). The results were consistent with our original findings showing metabolic multimorbidity was associated with an increase in the number of outpatient visits (IRR = 1.30) and days of hospitalization (IRR = 1.52). Similarly, the days of hospitalization increased substantially with age. Female patients used both the outpatient and inpatient care service more frequently than male patients. People with a higher economic level and those patients in economically underdeveloped regions had more days in hospital compared with their counterparts.

## Discussion

This study used nationally representative data to assess the prevalence and impact of metabolic multimorbidity among middle-aged and elderly Chinese adults. It was found that metabolic multimorbidity was common, especially among elderly participants and those living in urban areas. We identified that metabolic multimorbidity was positively associated a greater use of both outpatient and inpatient care utilization, as well as greater health expenditure for outpatient care. Moreover, we found that metabolic multimorbidity was positively associated with a higher likelihood of having functional difficulties (ADL limitation). However, we did not identify a positive association between metabolic multimorbidity and depression.

This study showed that 30.5% of persons aged ≥45 years have metabolic multimorbidity in China. Based on previous studies, the overall prevalence of metabolic syndrome was 16.5% in 2000 (29) and 23.3% in 2009 (7) among the Chinese adults. A meta-analysis study in 2016 revealed that the pooled estimate of metabolic syndrome prevalence was 24.5% among subjects in Mainland China (30). It is indicated that China is experiencing an emerging epidemic of metabolic syndrome, which might be related to accelerating changes in lifestyle and nutrition caused by rapid economic development and urbanization (31). The increasing prevalence of metabolic multimorbidity in developing countries may eventually become similar to that in developed countries. Several studies have reported a high prevalence of metabolic syndrome in United States (35%), Turkey (44%), and Iran (37%) (32–34). Variations in the prevalence of metabolic syndrome from different studies might be either due to real disparities in different countries or regions, or because of potential differences in the definitions, methods of data collection, and sampling of study populations (34).

Our results indicated that metabolic multimorbidity was positively associated with older age, urban area, and health insurance. Consistent with other studies (35, 36), the prevalence of metabolic multimorbidity increased with age, which can be attributed to the declining metabolic function during the aging process. Additionally, individuals living in urban areas are more likely to suffer from metabolic multimorbidity than those living in rural areas, which is in line with previous findings (30, 37). This may be attributable to the impact of urbanization, including unhealthy dietary patterns, decreased physical activity, uptake of a high caloric diet, excessive intake of fat and salt, all of which lead to the development of metabolic conditions (37–39). Notably, this study provides new evidence on the association between social health insurance and metabolic multimorbidity in China. People covered by social health insurance were more likely to suffer from metabolic multimorbidity, compared with individuals without health insurance.

Previous studies have demonstrated that multimorbidity is significantly associated with greater healthcare utilization and higher level of health expenditure, which has been well-documented in developed countries (40, 41). Similarly, our study revealed that individuals with metabolic multimorbidity were more likely to have more frequent outpatient visits and more days of hospitalization, as well as to spend more out-of-pocket money on outpatient care. However, we found there was no significant association between metabolic multimorbidity and OOPE for inpatient care. The reason may be that individuals with metabolic multimorbidity have fewer complications than patients with other patterns of multimorbidity (e.g., cardiovascular diseases, musculoskeletal diseases, and respiratory diseases), so they require fewer health services and spend less money on inpatient care at the early stage of disease. Therefore, prioritizing health and medical resource allocation will be needed to prevent and control metabolic multimorbidity and the complications in China (37). Social health insurance should play a greater role in financial risk protection by reducing OOPE on healthcare. Expanded insurance coverage and improved benefits packages for individuals with metabolic multimorbidity are warranted.

Existing studies have revealed that multimorbidity has a significantly negative effect on physical and mental health outcomes (15–17). However, our findings indicated that individuals with co-existing metabolic diseases were more likely to have ADL limitation, but metabolic multimorbidity was not significantly associated with IADL limitation and depression. This may be explained by that metabolic multimorbidity is less likely to impair the instrumental activities and mental health of patients than other patterns of multimorbidity. Even so, metabolic diseases have been demonstrated to play a dominant role in multiple multimorbidity patterns (39). Among multimorbidity groups, the musculoskeletal group, as well as the cardiovascular and metabolic groups, were identified as having a significant risk of ADL limitation (16). It has been documented that the association between metabolic syndrome and negative cardiovascular outcomes or mortality (42). Our study provides new evidence that significant associations of metabolic multimorbidity with functional limitations are found among in rural areas rather than urban areas.

The literature on the impact of multimorbidity on healthcare utilization and spending among individuals with metabolic diseases is relatively limited in developing countries. This is the first nationally representative study that examined the effect of metabolic multimorbidity on health service use, costs and health outcomes in China, by using metabolic biomarkers for the disease diagnosis. However, our study has several limitations. First, we examined the effect of multimorbidity by simply counting the number of chronic conditions without accounting for the different clusters and severity of chronic diseases, hence the accuracy of the findings may be affected for the types of metabolic multimorbidity. Second, this study is a cross-sectional design, so it is difficult to demonstrate the causal relationship between metabolic multimorbidity and healthcare utilization and spending. Third, this study only included middle-aged and older populations due to unavailable data of younger populations, which may exaggerate the prevalence of metabolic multimorbidity and its impacts.

In conclusion, metabolic multimorbidity has become a huge public health challenge to individuals and healthcare systems in China and other developing countries. There is a growing need to provide effective services to counter the impact of chronic metabolic multimorbidity. Targeting strategies and measures must be taken to control and reduce the increasing prevalence of metabolic multimorbidity. Healthcare systems need to shift from single-disease models to integrated care models to more effectively manage metabolic diseases and multimorbidity. Prioritizing health and medical resources allocation is needed to prevent, screen, and treat metabolic multimorbidity in the future.

## Data Availability Statement

The datasets presented in this study can be found in online repositories. The names of the repository/repositories and accession number(s) can be found at: The datasets generated and analyzed during the current study are available in the China Health and Retirement Longitudinal Study repository. http://charls.pku.edu.cn/pages/data/111/en.html.

## Author Contributions

YZ and HW conceived, designed the study, and wrote the first draft of the paper. YZ did the initial analysis. HW supervised data analysis. PZ, BO, JL, AH, TH, and HW critically revised the first draft. All authors reviewed and approved the final manuscript submitted for publication.

## Conflict of Interest

The authors declare that the research was conducted in the absence of any commercial or financial relationships that could be construed as a potential conflict of interest.
