# Supplementary material for: The Prevalence of Metabolic Disease Multimorbidity and Its Associations With Spending and Health Outcomes in Middle-Aged and Elderly Chinese Adults
Source: Front Public Health. 2021 May 3;9:658706. doi: 10.3389/fpubh.2021.658706 (PMC8126686; doi:10.3389/fpubh.2021.658706)
Supplement: Supplementary file 1 [file Data_Sheet_1.docx]

**Appendixes**

**Table S1.** The association between metabolic multimorbidity and the frequency of health service utilization

| **Variable (reference)** | **Number of outpatient visits** | | | |  | **Days of inpatient care** | | | |
| --- | --- | --- | --- | --- | --- | --- | --- | --- | --- |
|  | IRR | 95% CI | | P value |  | IRR | 95% CI | | P value |
| **Multimorbidity** (single disorder) | 1.30 | 1.04 | 1.62 | 0.021 |  | 1.50 | 1.26 | 1.78 | <0.001 |
| **Age** (45-59 years) |  |  |  |  |  |  |  |  |  |
| 55-65 | 0.98 | 0.79 | 1.22 | 0.868 |  | 1.53 | 1.23 | 1.91 | <0.001 |
| 65-75 | 1.09 | 0.83 | 1.41 | 0.543 |  | 1.91 | 1.51 | 2.42 | <0.001 |
| ≥75 | 1.07 | 0.74 | 1.55 | 0.704 |  | 2.50 | 1.86 | 3.34 | <0.001 |
| **Gender** (male) | 1.49 | 1.20 | 1.85 | <0.001 |  | 1.22 | 1.02 | 1.47 | 0.029 |
| **Marital status** (married) | 0.96 | 0.74 | 1.25 | 0.781 |  | 0.94 | 0.74 | 1.18 | 0.574 |
| **Education level** (Illiterate) |  |  |  |  |  |  |  |  |  |
| Primary school | 0.99 | 0.76 | 1.29 | 0.960 |  | 1.13 | 0.92 | 1.39 | 0.253 |
| Secondary school | 0.91 | 0.69 | 1.21 | 0.535 |  | 1.16 | 0.90 | 1.50 | 0.240 |
| College and above | 1.02 | 0.68 | 1.52 | 0.936 |  | 0.95 | 0.67 | 1.35 | 0.779 |
| **Residence place** (urban) | 1.23 | 0.97 | 1.55 | 0.093 |  | 1.07 | 0.89 | 1.28 | 0.462 |
| **Region (**east**)** |  |  |  |  |  |  |  |  |  |
| Central | 1.05 | 0.82 | 1.36 | 0.681 |  | 1.41 | 1.11 | 1.80 | 0.006 |
| West | 1.23 | 0.95 | 1.60 | 0.114 |  | 1.72 | 1.35 | 2.20 | <0.001 |
| **PCE** (<5000 RMB) |  |  |  |  |  |  |  |  |  |
| ≥5000 RMB | 1.11 | 0.88 | 1.39 | 0.385 |  | 1.50 | 1.24 | 1.80 | <0.001 |
| Missing | 1.09 | 0.86 | 1.38 | 0.456 |  | 1.31 | 1.09 | 1.59 | 0.005 |
| **Social health insurance** (none) | 1.19 | 0.89 | 1.59 | 0.249 |  | 1.14 | 0.90 | 1.44 | 0.270 |

**Notes:** Poisson regression models were used and adjusted for all socio-demographic covariates. IRR, incident rate ratio; CI, confidence interval; PCE, Per capita household annual consumption expenditure.

**Table S2.** The association of metabolic multimorbidity with healthcare expenditure in China.

| **Variable (reference)** | **OOPE for outpatient care** | | | |  | | **OOPE for inpatient care** | | | | |
| --- | --- | --- | --- | --- | --- | --- | --- | --- | --- | --- | --- |
|  | β | 95% CI | | P value |  | | β | | 95% CI | | P value |
| **Multimorbidity** (single disorder) | 0.226 | 0.054 | 0.397 | 0.010 |  | 0.044 | | -0.118 | | 0.205 | 0.596 |
| **Age** (45-59 years) |  |  |  |  |  |  | |  | |  |  |
| 55-65 | 0.120 | -0.103 | 0.343 | 0.291 |  | -0.107 | | -0.331 | | 0.117 | 0.350 |
| 65-75 | -0.010 | -0.255 | 0.236 | 0.939 |  | -0.245 | | -0.479 | | -0.011 | 0.040 |
| ≥75 | 0.232 | -0.141 | 0.605 | 0.222 |  | -0.215 | | -0.524 | | 0.093 | 0.171 |
| **Gender** (male) | 0.117 | -0.069 | 0.303 | 0.218 |  | -0.005 | | -0.174 | | 0.165 | 0.957 |
| **Marital status** (married) | -0.067 | -0.335 | 0.201 | 0.625 |  | -0.306 | | -0.543 | | -0.069 | 0.011 |
| **Education level** (Illiterate) |  |  |  |  |  |  | |  | |  |  |
| Primary school | 0.151 | -0.075 | 0.378 | 0.191 |  | -0.100 | | -0.305 | | 0.104 | 0.335 |
| Secondary school | -0.015 | -0.273 | 0.243 | 0.910 |  | -0.112 | | -0.344 | | 0.120 | 0.342 |
| College and above | -0.112 | -0.442 | 0.217 | 0.504 |  | -0.048 | | -0.355 | | 0.259 | 0.759 |
| **Residence place** (urban) | 0.061 | -0.126 | 0.247 | 0.525 |  | -0.066 | | -0.234 | | 0.102 | 0.442 |
| **Region (**east**)** |  |  |  |  |  |  | |  | |  |  |
| Central | 0.116 | -0.084 | 0.315 | 0.257 |  | -0.330 | | -0.519 | | -0.140 | 0.001 |
| West | -0.226 | -0.448 | -0.004 | 0.046 |  | -0.458 | | -0.666 | | -0.250 | 0.000 |
| **PCE** (<5000 RMB) |  |  |  |  |  |  | |  | |  |  |
| ≥5000 RMB | 0.579 | 0.374 | 0.785 | 0.000 |  | 0.532 | | 0.338 | | 0.725 | 0.000 |
| Missing | 0.243 | 0.028 | 0.457 | 0.027 |  | 0.134 | | -0.071 | | 0.339 | 0.201 |
| **Social health insurance** (none) | -0.007 | -0.269 | 0.255 | 0.959 |  | -0.052 | | -0.294 | | 0.190 | 0.672 |

Notes: The analyses were based on generalized linear models with a logarithm transfer. OOPE, Out-of-Pocket Expenditure; PCE, per capita household consumption expenditure; CI, confidence interval. The analyses were based on generalized linear models with a logarithm transfer.

**Table S3.** The association of metabolic multimorbidity with the frequency of healthcare utilization.

| **Variable (reference)** | **Number of outpatient visits** | | | |  | **Days of inpatient care** | | | |
| --- | --- | --- | --- | --- | --- | --- | --- | --- | --- |
|  | IRR | 95% CI | | P value |  | IRR | 95% CI | | P value |
| **Multimorbidity** (single disorder) | 1.30 | 1.05 | 1.61 | 0.017 |  | 1.52 | 1.28 | 1.81 | <0.001 |
| **Age** (45-59 years) |  |  |  |  |  |  |  |  |  |
| 55-65 | 0.97 | 0.78 | 1.22 | 0.818 |  | 1.55 | 1.25 | 1.93 | <0.001 |
| 65-75 | 1.09 | 0.85 | 1.39 | 0.484 |  | 1.99 | 1.57 | 2.53 | <0.001 |
| ≥75 | 1.11 | 0.75 | 1.64 | 0.609 |  | 2.58 | 1.94 | 3.42 | <0.001 |
| **Gender** (male) | 1.48 | 1.20 | 1.84 | <0.001 |  | 1.22 | 1.02 | 1.45 | 0.031 |
| **Marital status** (married) | 0.98 | 0.76 | 1.26 | 0.845 |  | 0.95 | 0.76 | 1.18 | 0.624 |
| **Education level** (Illiterate) |  |  |  |  |  |  |  |  |  |
| Primary school | 0.98 | 0.74 | 1.29 | 0.876 |  | 1.12 | 0.91 | 1.38 | 0.266 |
| Secondary school | 0.92 | 0.69 | 1.22 | 0.546 |  | 1.17 | 0.90 | 1.51 | 0.243 |
| College and above | 1.01 | 0.68 | 1.50 | 0.963 |  | 0.93 | 0.66 | 1.30 | 0.657 |
| **Residence place** (urban) | 1.21 | 0.96 | 1.52 | 0.107 |  | 1.07 | 0.89 | 1.29 | 0.460 |
| **Region (**east**)** |  |  |  |  |  |  |  |  |  |
| Central | 1.06 | 0.84 | 1.35 | 0.608 |  | 1.43 | 1.12 | 1.83 | 0.004 |
| West | 1.22 | 0.93 | 1.58 | 0.146 |  | 1.81 | 1.43 | 2.30 | <0.001 |
| **PCE** (<5000 RMB) |  |  |  |  |  |  |  |  |  |
| ≥5000 RMB | 1.10 | 0.88 | 1.38 | 0.401 |  | 1.52 | 1.27 | 1.82 | <0.001 |
| Missing | 1.10 | 0.86 | 1.40 | 0.448 |  | 1.34 | 1.10 | 1.61 | 0.003 |
| **Social health insurance** (none) | 1.13 | 0.83 | 1.53 | 0.449 |  | 1.15 | 0.90 | 1.46 | 0.268 |

Notes: Negative binomial regression models were used and adjusted for all socio-demographic covariates. IRR, incident rate ratio; CI, confidence interval; PCE, Per capita household annual consumption expenditure.

**Table S4.** The association of metabolic multimorbidity with healthcare expenditure in China.

| **Variable (reference)** | **OOPE for outpatient care** | | | |  | | **OOPE for inpatient care** | | | | |
| --- | --- | --- | --- | --- | --- | --- | --- | --- | --- | --- | --- |
|  | β | 95% CI | | P value |  | | β | | 95% CI | | P value |
| **Multimorbidity** (single disorder) | 82.99 | 17.70 | 148.27 | 0.013 |  | 108.43 | | -457.28 | | 674.13 | 0.707 |
| **Age** (45-59 years) |  |  |  |  |  |  | |  | |  |  |
| 55-65 | -15.82 | -91.76 | 60.12 | 0.682 |  | 556.90 | | -44.11 | | 1157.92 | 0.069 |
| 65-75 | 3.11 | -76.74 | 82.95 | 0.939 |  | 1069.96 | | 1.40 | | 2138.53 | 0.050 |
| ≥75 | -95.19 | -209.34 | 18.96 | 0.102 |  | 709.60 | | 92.09 | | 1327.12 | 0.024 |
| **Gender** (male) | 47.19 | 2.41 | 91.97 | 0.039 |  | 498.08 | | -198.28 | | 1194.45 | 0.161 |
| **Marital status** (married) | 60.30 | -99.48 | 220.08 | 0.459 |  | -631.38 | | -1081.46 | | -181.30 | 0.006 |
| **Education level** (Illiterate) |  |  |  |  |  |  | |  | |  |  |
| Primary school | 36.39 | -27.49 | 100.26 | 0.263 |  | 663.58 | | -237.96 | | 1565.11 | 0.149 |
| Secondary school | 12.45 | -55.44 | 80.34 | 0.719 |  | 211.25 | | -345.17 | | 767.67 | 0.456 |
| College and above | -21.02 | -86.50 | 44.47 | 0.529 |  | 224.85 | | -1138.43 | | 1588.13 | 0.746 |
| **Residence place** (urban) | 98.08 | 27.45 | 168.70 | 0.007 |  | -238.30 | | -701.73 | | 225.14 | 0.313 |
| **Region (**east**)** |  |  |  |  |  |  | |  | |  |  |
| Central | -5.20 | -75.37 | 64.97 | 0.884 |  | -321.03 | | -1043.33 | | 401.28 | 0.383 |
| West | -34.89 | -120.00 | 50.23 | 0.421 |  | -303.85 | | -902.61 | | 294.92 | 0.319 |
| **PCE** (<5000 RMB) |  |  |  |  |  |  | |  | |  |  |
| ≥5000 RMB | 128.44 | 69.77 | 187.12 | <0.001 |  | 1218.25 | | 530.10 | | 1906.40 | 0.001 |
| Missing | 84.70 | 13.44 | 155.96 | 0.020 |  | 166.34 | | -181.59 | | 514.27 | 0.348 |
| **Social health insurance** (none) | 10.89 | -59.80 | 81.57 | 0.762 |  | 526.56 | | 25.51 | | 1027.62 | 0.039 |

Notes: Multivariable linear models were used. OOPE, Out-of-Pocket Expenditure; PCE, per capita household consumption expenditure; CI, confidence interval.

**Table S5**. The association of metabolic multimorbidity with functional and mental health.

| **Variable (reference)** | **ADL limitation** | | | | **IADL limitation** | | | | **Depression** | | | |
| --- | --- | --- | --- | --- | --- | --- | --- | --- | --- | --- | --- | --- |
|  | AOR | 95% CI | | P value | AOR | 95% CI | | P value | AOR | 95% CI | | P value |
| **Multimorbidity** (single disorder) | 1.36 | 1.18 | 1.57 | <0.001 | 1.16 | 1.00 | 1.34 | 0.051 | 0.99 | 0.84 | 1.16 | 0.871 |
| **Age** (45-59 years) |  |  |  |  |  |  |  |  |  |  |  |  |
| 55-65 | 1.47 | 1.05 | 2.04 | 0.023 | 1.61 | 1.18 | 2.18 | 0.003 | 1.59 | 1.28 | 1.98 | <0.001 |
| 65-75 | 2.15 | 1.72 | 2.68 | <0.001 | 2.50 | 2.02 | 3.10 | <0.001 | 1.87 | 1.34 | 2.59 | <0.001 |
| ≥75 | 2.94 | 1.98 | 4.39 | <0.001 | 4.80 | 3.30 | 6.98 | <0.001 | 0.99 | 0.71 | 1.37 | 0.940 |
| **Gender** (male) | 1.45 | 1.18 | 1.77 | <0.001 | 1.90 | 1.57 | 2.30 | <0.001 | 2.03 | 1.74 | 2.36 | <0.001 |
| **Marital status** (married) | 1.11 | 0.87 | 1.42 | 0.392 | 0.93 | 0.75 | 1.16 | 0.508 | 1.31 | 1.08 | 1.58 | 0.006 |
| **Education level** (Illiterate) |  |  |  |  |  |  |  |  |  |  |  |  |
| Primary school | 0.72 | 0.56 | 0.92 | 0.008 | 0.49 | 0.39 | 0.62 | <0.001 | 0.81 | 0.67 | 0.98 | 0.033 |
| Secondary school | 0.62 | 0.50 | 0.78 | <0.001 | 0.48 | 0.38 | 0.60 | <0.001 | 0.87 | 0.67 | 1.13 | 0.294 |
| College and above | 0.37 | 0.23 | 0.59 | <0.001 | 0.21 | 0.13 | 0.34 | <0.001 | 0.43 | 0.32 | 0.59 | <0.001 |
| **Residence place** (urban) | 1.37 | 1.07 | 1.73 | 0.011 | 1.46 | 1.17 | 1.83 | 0.001 | 1.78 | 1.49 | 2.12 | <0.001 |
| **Region (**east**)** |  |  |  |  |  |  |  |  |  |  |  |  |
| Central | 1.81 | 1.41 | 2.31 | <0.001 | 1.73 | 1.37 | 2.18 | <0.001 | 1.49 | 1.23 | 1.80 | <0.001 |
| West | 1.46 | 1.12 | 1.92 | 0.006 | 1.70 | 1.31 | 2.19 | <0.001 | 1.86 | 1.51 | 2.28 | <0.001 |
| **PCE** (<5000 RMB) |  |  |  |  |  |  |  |  |  |  |  |  |
| ≥5000 RMB | 1.03 | 0.83 | 1.27 | 0.800 | 0.90 | 0.73 | 1.11 | 0.314 | 0.92 | 0.76 | 1.11 | 0.369 |
| Missing | 1.23 | 1.01 | 1.50 | 0.043 | 1.18 | 0.98 | 1.43 | 0.076 | 0.88 | 0.74 | 1.06 | 0.182 |
| **Social health insurance** (none) | 1.21 | 0.96 | 1.51 | 0.103 | 1.16 | 0.95 | 1.43 | 0.145 | 2.47 | 1.99 | 3.07 | <0.001 |

Notes: PCE, Per capita household annual consumption expenditure; AOR, Adjusted odds ratio; CI, confidence interval.
